# Vaccination Coverage Among Mothers and Close Contacts of Neonates Hospitalized in NICU in Southern Greece: A Cocooning Strategy Approach

**DOI:** 10.3390/vaccines14070637

**Published:** 2026-07-20

**Authors:** Angeliki Irakleous, Eleni Vergadi, Despoina Gkentzi, Eleftheria Hatzidaki

**Affiliations:** 1 Department of Neonatology/Neonatal Intensive Care Unit, University Hospital of Heraklion, School of Medicine, University of Crete, 70013 Heraklion, Greece; med9p1110074@med.uoc.gr; 2 Department of Paediatrics, University General Hospital of Heraklion, Medical School, University of Crete, 71500 Heraklion, Greece; eleni.vergadi@uoc.gr; 3 Department of Paediatrics, University General Hospital of Patras, Medical School, University of Patras, 26504 Rio, Greece; gkentzid@upatras.gr

**Keywords:** cocooning, neonatal intensive care unit (NICU), vaccination coverage, influenza, pertussis, caregiver immunization, maternal vaccination

## Abstract

**Background:** Neonates and young infants are susceptible to vaccine-preventable diseases because of immature immune responses and delayed acquisition of vaccine-induced protection. As household members and caregivers are a major source of exposure, international organizations recommend cocooning, i.e., vaccination of susceptible individuals who are in regular contact with infants, to provide complementary indirect neonatal protection. To evaluate cocooning implementation, we assessed influenza and pertussis vaccination uptake among mothers and close contacts of hospitalized neonates and identified factors associated with vaccine uptake. **Methods:** Mothers of neonates hospitalized in a Neonatal Intensive Care Unit (NICU) between 1 January 2022 and 31 December 2024 were invited to complete a structured questionnaire. Mothers reported their own vaccination status and that of the neonate’s close contacts. Associations between maternal characteristics and vaccine uptake were examined using logistic regression. **Results:** In total, 405 mothers participated. Influenza vaccination uptake was 47.6% among mothers, and at least one adult close contact was vaccinated in 53.6% of families. Pertussis vaccination uptake was 40.5% among mothers, and at least one adult close contact was vaccinated in 32.3% of families. The most frequently reported reason for non-vaccination was low perceived need among unvaccinated mothers (74.1% for influenza and 83.4% for pertussis) and among families with incompletely vaccinated adult close contacts (81.8% for influenza and 89.7% for pertussis). In multivariable analyses, higher maternal education was associated with increased uptake of both influenza (OR 2.36, 95% CI: 1.47–3.80, *p* < 0.001) and pertussis vaccination (OR 4.25, 95% CI 2.02–8.94, *p* < 0.001), whereas younger maternal age (≤25 years) was associated with lower influenza vaccine acceptance (OR 0.24, 95% Cl: 0.1–0.60, *p* = 0.002). **Conclusions:** Vaccination uptake among mothers and adult close contacts of NICU-admitted neonates was suboptimal for both influenza and pertussis vaccines. Targeted counseling before delivery, reinforced during maternity or NICU hospitalization as a catch-up opportunity, and system-level implementation measures aligned with the dominant barrier of low perceived need are needed to strengthen cocooning in this high-risk population.

## 1. Introduction

Neonates and young infants are vulnerable to infectious diseases during the first months of life due to incomplete maturation of innate and adaptive immune responses [1,2,3]. Reduced phagocytic function, attenuated Th1-polarized cytokine responses, suboptimal B-cell maturation, and limited generation of high-affinity antibodies collectively limit the ability of neonates to mount robust vaccine-induced immune responses [1,2,3]. The transplacental transfer of maternal IgG antibodies during pregnancy and the provision of immunomodulatory factors, including secretory IgA antibodies, through breastfeeding, constitute key defense mechanisms against infectious agents in early life [2,4,5,6,7,8]. For infections such as influenza and pertussis, infants rely heavily on maternally transferred antibodies for protection. In the absence of adequate maternal immunity, or among infants born prematurely, protection may be suboptimal until infants develop their own immune responses following vaccination [2,4]. Influenza and pertussis, along with other respiratory tract infections, continue to impose a substantial global public health burden [9,10]. Notably, infants younger than six months of age have the highest incidence of influenza-associated hospitalizations and the highest risk of influenza-associated mortality among children [9,11,12]. Similarly, the highest morbidity and mortality rates from *Bordetella pertussis* occur in infants younger than six months, especially those under two months of age [10,12,13]. At the same time, routine infant immunization schedules typically begin at two months of age for pertussis-containing vaccines and at six months of age for influenza vaccination [14]. Several epidemiological studies have identified family members as the main source of transmission of airborne pathogens to young infants. Parents account for approximately half of infections in infants younger than six months of age [15]. In most studies, mothers are the most frequent source, followed by fathers, siblings, and other caregivers [15]. In a study by Wiley et al. examining sources of pertussis transmission to infants, mothers were responsible for 39% of cases, fathers for 16%, and grandparents for 5% [16]. The contribution of siblings (16–43%) and non-household contacts (4–22%) varied considerably across studies [16]. In addition to maternal immunization, the cocooning strategy—vaccinating individuals who have regular contact with infants, including parents, siblings, grandparents, and other caregivers—is recommended to minimize the risk of pathogen transmission during early infancy [17].

International and national authorities, including the World Health Organization (WHO) [14], the Centers for Disease Control and Prevention (CDC) [18], and the Greek Ministry of Health [19], support maternal vaccination, while cocooning, vaccination of susceptible household contacts, is used as a complementary measure. Pregnant women are advised to receive the seasonal inactivated influenza vaccine at any gestational age, ideally before or early in the influenza season. Influenza vaccination is also recommended for postpartum and breastfeeding women who were not vaccinated during pregnancy, as well as for other eligible individuals who are in close contact with infants younger than six months of age. For pertussis, a single dose of Tdap or Tdap-IPV is recommended during each pregnancy, preferably between 27 and 36 weeks of gestation. Postpartum women who did not receive Tdap during pregnancy and household contacts who are not up to date with pertussis vaccination are also advised to be vaccinated. Importantly, inactivated influenza vaccines and Tdap are considered safe during pregnancy; influenza vaccination can be administered in any trimester, and available data do not indicate an increased frequency or unusual pattern of adverse events after Tdap vaccination [18].

Evidence supports that vaccination during pregnancy improves neonatal passive immunity by increasing maternal antibody levels and enhancing the transfer of protective antibodies to the fetus [20,21]. However, preterm infants may receive lower concentrations of maternally derived antibodies because transplacental antibody transfer occurs predominantly during the third trimester. Consequently, cocooning strategies may provide an important additional layer of protection for these particularly vulnerable infants [2].

The benefits of maternal immunization and cocooning strategies have been supported by numerous studies [22,23,24]. Surveillance data from the United States showed that influenza vaccination during pregnancy was associated with a 40% reduction in influenza-related hospitalizations among pregnant women and a 72% reduction among infants under six months of age [24]. Maternal pertussis vaccination also reduced pertussis incidence by 77.7% and pertussis-related hospitalization by 90.5% among infants younger than two months of age [24]. Halasa et al. reported that maternal vaccination against SARS-CoV-2 lowered the risk of hospitalization and severe disease among infants younger than six months [22]. Moreover, Oguz et al. reported that vaccination of household contacts was associated with lower rates of influenza-like illness and severe acute respiratory infection during early infancy [23]. Unfortunately, despite the scientific evidence, maternal and household immunization remains limited [25,26,27,28,29].

The present study aimed to record vaccination coverage against influenza and pertussis among mothers and close contacts of neonates admitted to the NICU and to evaluate demographic, clinical, and behavioral factors associated with vaccine uptake. Because NICU-admitted neonates represent a particularly vulnerable group, the study focused on cocooning implementation in this high-risk setting rather than estimating vaccination coverage in the general mother–baby population.

## 2. Materials and Methods

A cross-sectional, questionnaire-based study was conducted among postpartum women whose newborns were admitted to the Neonatal Department/Neonatal Intensive Care Unit (NICU) of the University General Hospital of Heraklion, Crete, Greece, between 1 January 2022, and 31 December 2024.

Data were collected using a structured questionnaire developed by the study investigators for the purposes of the study (Supplementary File S1). After providing verbal consent, mothers completed the questionnaire via telephone interview. Interviews were conducted after approval by the Scientific Council of the University General Hospital of Heraklion (Protocol Number: 25272/01-08-2025; Approval Date: 13 August 2025).

Mothers were eligible for participation if their newborn had been hospitalized in the NICU during the study period, they were reachable by telephone, and they provided verbal consent to participate. Mothers who declined participation, could not be reached by telephone, or were unable to complete the interview or communicate in Greek or English were excluded from the study.

The questionnaire contained items on demographic characteristics, pregnancy-related information, vaccination uptake among mothers and close contacts, and reasons for non-vaccination. Close contacts included all family members and caregivers who regularly interacted with the infant. The high-risk group for influenza included individuals with chronic medical conditions, such as immune suppression, chronic respiratory diseases, cardiovascular diseases, and/or diabetes mellitus. The questionnaire did not systematically record whether a healthcare professional had recommended vaccination during pregnancy; therefore, this factor was not included in the statistical analyses.

Maternal vaccination status and that of the neonate’s close contacts were based on maternal report during the telephone interview. Postpartum vaccination was defined as vaccination after the index delivery and before the interview; exact vaccination dates were not collected. Close contacts were considered vaccinated against pertussis if they had received a pertussis-containing vaccine within the previous 10 years.

### Statistical Analysis

The sample size required was calculated considering that at least 300 mothers were needed to achieve 80% statistical power at a significance level of α = 0.05 for estimating vaccination coverage.

Descriptive statistical analysis was performed for all variables. Categorical variables are presented as absolute (*n*) and relative (%) frequencies, whereas continuous variables were assessed for normality and are presented as mean ± standard deviation (SD) or median (range), as appropriate. Occupational status was recorded into four categories (education, healthcare professionals, domestic/unemployed, and other professions). Pearson’s chi-square (χ^2^) test or Fisher’s exact test, as appropriate, was used for comparisons across the three study years. Binary logistic regression was performed to assess the association of demographic factors with maternal vaccination uptake. For the regression models, vaccination during pregnancy and postpartum vaccination were combined into a single “any vaccination” outcome to identify factors associated with overall uptake; descriptive results remained separated by timing. Because exact vaccination dates were unavailable, the composite outcome reflects uptake rather than the timeliness of vaccination in relation to pregnancy, delivery, or the period of greatest infant vulnerability. Odds ratios (ORs) with 95% confidence intervals (CIs) are reported. Season of delivery was described in Table 1 but was not included in the regression models because it is an imperfect proxy for vaccination timing and exact dates relative to the influenza season were unavailable. Statistical significance was set at 5%. Analyses were performed using IBM SPSS Statistics, version 26.0.

## 3. Results

### 3.1. Demographic Characteristics

A total of 1236 mothers were eligible for inclusion. Telephone contact was attempted for 801 mothers: 405 participated, 123 declined, and 273 could not be reached. The participation proportion among all mothers for whom contact was attempted was 50.6% (405/801), and the participation proportion among those successfully reached was 76.7% (405/528). Recruitment was stopped once the required sample size had been achieved; therefore, the remaining 435 eligible mothers were not contacted. Participants were evenly distributed across the study period, with 135 mothers enrolled in each year (2022, 2023, and 2024).

Regarding maternal age, 44.7% (181/405) of mothers were aged ≥35 years, 29.6% (120/405) were 30–34 years old, 14.1% (57/405) were 26–29 years old, and 11.6% (47/405) were ≤25 years old. Most participants were of Greek origin (374/405, 92.3%), with smaller proportions reporting Albanian, Bulgarian, or other nationalities. Educational level was mainly secondary education (220/405, 54.3%), followed by tertiary education (120/405, 29.6%), postgraduate/doctoral studies (43/405, 10.6%), and primary education (22/405, 5.4%). Regarding professional status, approximately half of the women belonged to the category “other professions” (196/405, 48.4%), one third were domestic/unemployed (134/405, 33.1%), and smaller proportions worked in education (42/405, 10.4%) or were healthcare professionals (33/405, 8.1%). Women who met criteria for influenza high-risk status represented 8.9% (36/405) of the cohort. Most pregnancies were singleton (373/405, 92.1%), and most conceptions occurred naturally (350/405, 86.4%). Assisted reproduction accounted for 13–14% of pregnancies each year. All participants reported regular medical follow-up during pregnancy.

Overall, the enrolled mothers had 436 neonates admitted to the NICU during the study period (145 were born in 2022, 145 in 2023, and 146 in 2024). The proportion of premature and full-term newborns remained comparable during the three-year period. Specifically, 216 newborns were premature (69 in 2022, 68 in 2023, and 79 in 2024) and 220 were full-term (76 in 2022, 77 in 2023, and 67 in 2024). Season of delivery is shown descriptively in Table 1. The demographic characteristics of mothers and their neonates are summarized in Table 1.

### 3.2. Maternal Vaccination During Pregnancy and the Postpartum Period

We classified maternal vaccination status for influenza and pertussis in three groups: no vaccination, vaccination during pregnancy, and vaccination during the postpartum period.

For influenza vaccination, approximately half of the women in each year (48.1%, 54.8%, and 54.1% in 2022, 2023, and 2024, respectively) remained unvaccinated during pregnancy and postpartum, while a stable proportion received the vaccine only after delivery (11.9–14.8%). Overall maternal vaccination coverage (including postpartum vaccination) remained stable over the three-year period (51.9%, 45.2%, and 45.9% in 2022, 2023, and 2024, respectively), without significant variation during the period under study.

Pertussis vaccination status improved over time. At the beginning of the three-year period, most women (71.9%, 97/135) remained unvaccinated during pregnancy and postpartum, while 16.3% (22/135) reported vaccination during pregnancy and 11.9% (16/135) after delivery. In the following years, vaccination uptake increased (38.5% of women were vaccinated in 2023 and 54.8% in 2024), with increases in the proportions of women vaccinated during pregnancy and postpartum, while the percentage of fully unvaccinated women gradually decreased (61.5% in 2023 and 45.2% in 2024). Table 2 presents influenza and pertussis vaccination uptake during pregnancy and the postpartum period by study year.

### 3.3. Reasons for Non-Vaccination During Pregnancy and the Postpartum Period

Regarding influenza immunization, the main barrier to vaccination was the perception that vaccination was unnecessary (157/212, 74.1%). Safety concerns were reported less frequently (38/212, 17.9%), although they showed a modest rise over time. Similarly, among women who did not receive the pertussis vaccine, the majority (201/241, 83.4%) stated that vaccination was unnecessary, while safety concerns (19/241, 7.9%) and a general negative attitude towards vaccination (21/241, 8.7%) were less common reasons. Reasons for non-vaccination for both vaccines are described in Table 3.

### 3.4. Vaccination Coverage Among Close Contacts of Neonates and Reasons for Non-Vaccination

Vaccination status among adult close contacts of the neonates (fathers and other close caregivers) and siblings is presented in Table 4. Because mothers reported household-level categories rather than the number and vaccination status of each individual contact, the adult-contact estimates are expressed at the family level.

For influenza, 46.4% (188/405) of families reported that no adult close contact was vaccinated, whereas 34.8% (141/405) reported that all adult close contacts were vaccinated. At least one adult close contact was vaccinated in 53.6% (217/405) of families. Among families with siblings, reported influenza vaccination among siblings ranged from 45.3% to 55.4% across the three study years (overall 51.9%, 112/216).

Pertussis vaccination among adult close contacts was lower than influenza vaccination but improved over the three-year study period. In 2022, 79.3% (107/135) of families reported that no adult close contact had been vaccinated, and 4.4% (6/135) reported that all adult close contacts had been vaccinated. By 2024, the proportion reporting that all adult close contacts were vaccinated had increased to 20.7% (28/135), while the proportion reporting that none were vaccinated had decreased to 54.1% (73/135). The proportion in the “father only” category also increased over time.

Reasons for non-vaccination among close contacts are presented in Table 5. Among families in which adult contacts were not all vaccinated, low perceived need was the most frequently reported reason for non-vaccination against influenza (81.8%, 216/264) and pertussis (89.7%, 321/358). Among unvaccinated siblings, low perceived need reported by parents was also the leading reason for influenza non-vaccination (78.8%, 82/104). Safety concerns and general vaccine hesitancy were reported less frequently.

### 3.5. Subsequent Pregnancies and Adherence to NICU Recommendations

Only a small proportion of women reported having another child during the following years, a fact that should be considered when interpreting these results. In total, 57 women (of 405) reported a subsequent pregnancy. Among them, only 30/57 (52.6%) reported following, either partially or fully, the NICU staff recommendations for influenza and pertussis vaccination (Table 6).

### 3.6. Association Between Vaccination and Demographic Characteristics

Education was strongly associated with vaccine uptake for both influenza and pertussis (Table 7 and Table 8). Women with tertiary education were approximately twice as likely to have received at least one dose of influenza vaccine during pregnancy and/or the postpartum period compared with those with secondary education (OR 2.36, 95% CI: 1.47–3.80, *p* < 0.001). For pertussis, the effect of educational level was even more pronounced at higher levels of education. Women with postgraduate/doctoral studies were approximately four times more likely to be vaccinated against pertussis compared with women with secondary education (OR 4.25, 95% CI: 2.02–8.94, *p* < 0.001). In contrast, women aged ≤25 years showed a significantly lower probability of influenza vaccination compared with the reference group aged 26–29 years (OR 0.24, 95% CI: 0.10–0.60, *p* = 0.002).

## 4. Discussion

This study recorded the vaccination coverage among mothers and close contacts of neonates hospitalized in the Neonatology Department/Neonatal Intensive Care Unit of the University General Hospital of Heraklion, Crete, Greece, between 1 January 2022 and 31 December 2024. The study population should be interpreted as a NICU-based, high-risk cohort rather than a general mother–infant population.

Most participants were Greek (92.3%), and approximately half were aged 35 years or older. According to national data, a shift toward advanced maternal age has been observed in recent years in Greece [30]. The increased proportion of pregnant women aged ≥35 years is probably attributable to socioeconomic factors, delayed childbearing, and wider use of assisted reproductive technologies [30].

Maternal vaccination uptake and family-level vaccination among adult close contacts were suboptimal. Influenza vaccination uptake was 47.6% among mothers, and at least one adult close contact was vaccinated in 53.6% of families. Pertussis vaccination uptake was 40.5% among mothers, and at least one adult close contact was vaccinated in 32.3% of families. The primary reason for non-vaccination was low perceived need among both mothers and families with incompletely vaccinated contacts. Higher educational attainment was associated with increased vaccine uptake, while women aged ≤25 years had lower influenza vaccination uptake.

Our findings show improved uptake compared with earlier Greek reports but remain below desirable targets. Gkentzi et al. reported that only 6% of pregnant women were immunized against influenza and pertussis during pregnancy [25]. More recent findings by Maltezou et al. reported influenza coverage up to 45.7% and pertussis coverage up to 16.8% during pregnancy [26]. In our cohort, pertussis vaccination increased over the three-year period, whereas influenza coverage remained practically stable (around 45–52%), despite longstanding influenza recommendations and more recent efforts to strengthen pertussis vaccination during pregnancy.

A recent systematic review of studies published between 2000 and 2023 confirmed that maternal influenza and pertussis vaccination coverage varies widely across countries and remains suboptimal in many settings [31]. Previous studies also underscore wide variability in cocooning implementation. A study from India reported higher maternal coverage for influenza (up to 64.1%) and pertussis (48.5%) among mothers of preterm infants [27]. Likewise, in a French study, maternal coverage for pertussis reached 66.8% [28]. In addition, analyses of high-performing pregnancy vaccination programs in the United Kingdom, the United States, and Spain suggest that high coverage depends on national recommendations, vaccine reimbursement, integration into antenatal care, healthcare-professional mobilization, and targeted educational material [32]. These findings provide useful benchmarks for interpreting the lower coverage observed in our cohort.

Across all three years, the primary reason for vaccine hesitancy among unvaccinated mothers was the perception that vaccination was “unnecessary”, while safety concerns and fear of adverse effects were reported less frequently. Consistent with international evidence, maternal vaccine hesitancy is commonly driven by doubts about vaccine effectiveness, limited awareness that vaccination is recommended during every pregnancy, and concerns regarding vaccine safety [33]. These findings support the need for counseling that explicitly addresses disease severity in early infancy, vaccine safety during pregnancy, and the indirect protection provided by cocooning.

The association between higher educational attainment and vaccine uptake, together with the lower influenza uptake observed among women aged ≤25 years, is consistent with previous studies [26,34]. Similar findings have been reported in Crete, the Greek island where our NICU is located: higher educational attainment and older maternal age were associated with influenza vaccination during pregnancy, whereas pertussis vaccination was mainly associated with educational attainment [35].

Although our questionnaire did not systematically capture whether vaccination had been recommended by a healthcare professional, previous studies emphasize the crucial role of healthcare professionals, particularly obstetricians, in promoting maternal immunization and cocooning [36]. Physician recommendation and awareness of disease severity during pregnancy have been associated with increased vaccination acceptance [26]. Moreover, low birth weight and larger household size have been related to higher vaccine uptake [29], whereas socioeconomic barriers, safety concerns, and absence of professional recommendation have been linked with hesitancy [33,37].

In our study, vaccination coverage among fathers and other caregivers for both influenza and pertussis was insufficient. In many cases, implementation of the cocooning strategy was limited to the father and was not extended to other household members and caregivers. This highlights the need for organized and targeted interventions to improve vaccine uptake among all individuals in close contact with vulnerable infants. Notably, a hospital-based program implemented during the 2010 pertussis epidemic in California demonstrated that offering Tdap vaccine during maternity hospitalization significantly increased vaccination coverage among close contacts of neonates (84.8% vs. 52.2% in the control group) and improved overall household uptake (76% vs. 29.3%) [38].

As observed among mothers, the predominant reason for non-vaccination among household contacts was low perceived need for vaccination. We did not collect socioeconomic factors or behavioral determinants that influence vaccine uptake in this study; therefore, we were unable to explore factors underlying vaccine hesitancy in this population. Previous studies have shown that paternal vaccination is strongly associated with maternal vaccination status; fathers are more likely to be vaccinated when their partners are vaccinated [29]. Seasonality may also influence the perceived need for vaccination, particularly for influenza, and future studies should incorporate exact vaccination dates, trimester at vaccination, and timing relative to the influenza season.

Our study has several limitations. First, it was a single-center study that included only mothers of infants admitted to a NICU, which limits generalizability; NICU admission may be associated with maternal, obstetric, neonatal, and vaccination-related factors; therefore, uptake in this cohort may differ from that in the general postpartum population. Second, data were collected through telephone interviews, a method that carries the possibility of recall and reporting bias. Vaccination status was self-reported for mothers and proxy-reported for household contacts, and records were not verified against official immunization registries. Third, a considerable proportion of eligible mothers could not be reached, raising the possibility of selection bias. Fourth, socioeconomic and other determinants of vaccination uptake were not collected, limiting our ability to identify factors associated with vaccination behavior. Fifth, although season of delivery was available, exact vaccination dates were not collected, preventing a seasonality-adjusted analysis. The timing of postpartum vaccination was also unavailable; therefore, we could not determine whether postpartum doses were administered early enough to protect infants during the period of greatest vulnerability. Finally, adult-contact data were collected as household-level categories rather than individual-level vaccination records, so contact estimates should not be interpreted as the proportion of all individual contacts vaccinated.

Vaccine hesitancy remains an important challenge [39]. More coordinated public health interventions are therefore necessary. Maternal vaccination coverage should be monitored at national and international levels, and interventions should be targeted to populations with low uptake. Education of healthcare professionals, especially obstetricians, neonatologists, and pediatricians, is essential and should address vaccine indications, mechanisms, effectiveness, and safety [36,40].

Parental education should emphasize the benefits of vaccination during pregnancy because maternal vaccination enhances passive immunity during the first months of life. Postpartum vaccination should be viewed as a catch-up strategy that may reduce subsequent household transmission but cannot provide the same immediate transplacental antibody protection as vaccination during pregnancy. Because exact postpartum vaccination dates were not available in our study, postpartum uptake cannot be equated with timely neonatal protection. Other strategies to optimize vaccination uptake could include broader public awareness campaigns and tools such as mobile health applications [41]. Prenatal counseling is particularly important because the period after delivery is often dominated by breastfeeding, newborn care, and adjustment to family routines, which may reduce opportunities for vaccine counseling and acceptance [42]. Nevertheless, offering vaccination during maternity hospitalization can increase uptake among mothers and household contacts, whereas delaying vaccination until after discharge is associated with lower acceptance [29,43].

## 5. Conclusions

Implementation of the cocooning strategy remains insufficient among families of NICU-admitted neonates in Southern Greece. Pertussis vaccination uptake improved over the three-year study period, whereas influenza vaccination uptake remained relatively stable. Because the most common barrier was low perceived need, counseling should begin during routine antenatal care and be reinforced during maternity or NICU hospitalization as a catch-up opportunity for mothers and close contacts. Coordinated obstetric, neonatal, and paediatric counseling, reminder systems, and hospital-based vaccination pathways may improve timely vaccine uptake and protection of young infants against vaccine-preventable diseases.

## Figures and Tables

**Table 1:** Demographic and obstetric characteristics of the study population by year of participation.

	Category	2022	2023	2024	Total
		*n* (%)	*n* (%)	*n* (%)	*n* (%)
Pregnancy type	Singleton pregnancy	125 (92.6%)	124 (91.9%)	124 (91.9%)	373 (92.1%)
	Twins	9 (6.7%)	11 (8.1%)	11 (8.1%)	31 (7.7%)
	Triplets	1 (0.7%)	0 (0.0%)	0 (0.0%)	1 (0.2%)
Age group (years)	≤25 years	13 (9.6%)	13 (9.6%)	21 (15.6%)	47 (11.6%)
	26–29 years	8 (5.9%)	22 (16.3%)	27 (20.0%)	57 (14.1%)
	30–34 years	45 (33.3%)	39 (28.9%)	36 (26.7%)	120 (29.6%)
	≥35 years	69 (51.1%)	61 (45.2%)	51 (37.8%)	181 (44.7%)
Nationality	Greek	123 (91.1%)	129 (95.6%)	122 (90.4%)	374 (92.3%)
	Other	12 (8.8%)	6 (4.4%)	13 (9.6%)	31 (7.6%)
Educational level	Primary education	10 (7.4%)	6 (4.4%)	6 (4.4%)	22 (5.4%)
	Secondary education	65 (48.1%)	74 (54.8%)	81 (60.0%)	220 (54.3%)
	Tertiary education	48 (35.6%)	39 (28.9%)	33 (24.4%)	120 (29.6%)
	Postgraduate/Doctoral studies	12 (8.9%)	16 (11.9%)	15 (11.1%)	43 (10.6%)
Occupational category	Education	11 (8.1%)	17 (12.6%)	14 (10.4%)	42 (10.4%)
	Healthcare professional	6 (4.4%)	12 (8.9%)	15 (11.1%)	33 (8.1%)
	Other professions	77 (57.0%)	59 (43.7%)	60 (44.4%)	196 (48.4%)
	Domestic/unemployed	41 (30.4%)	47 (34.8%)	46 (34.1%)	134 (33.1%)
Marital status	Married	118 (87.4%)	125 (92.6%)	124 (91.9%)	367 (90.6%)
	Unmarried	14 (10.4%)	7 (5.2%)	9 (6.7%)	30 (7.4%)
	Divorced/Widowed	3 (2.2%)	3 (2.2%)	1 (0.7%)	7 (1.7%)
	Unknown/ not reported	0 (0.0%)	0 (0.0%)	1 (0.7%)	1 (0.2%)
Number of children	1	37 (27.4%)	59 (43.7%)	53 (39.3%)	149 (36.8%)
	2	63 (46.7%)	47 (34.8%)	45 (33.3%)	155 (38.3%)
	3	24 (17.8%)	21 (15.6%)	24 (17.8%)	69 (17.0%)
	4	8 (5.9%)	7 (5.2%)	10 (7.4%)	25 (6.2%)
	≥5	3 (2.2%)	1 (0.7%)	3 (2.2%)	7 (1.7%)
High-risk group for influenza	No	122 (90.4%)	122 (90.4%)	125 (92.6%)	369 (91.1%)
	Yes	13 (9.6%)	13 (9.6%)	10 (7.4%)	36 (8.9%)
Health insurance coverage	No	7 (5.2%)	1 (0.7%)	2 (1.5%)	10 (2.5%)
	Yes	128 (94.8%)	134 (99.3%)	133 (98.5%)	395 (97.5%)
Mode of conception	Natural conception	116 (85.9%)	117 (86.7%)	117 (86.7%)	350 (86.4%)
	Assisted reproduction	19 (14.1%)	18 (13.3%)	18 (13.3%)	55 (13.6%)
Prenatal medical follow-up	Yes	135 (100%)	135 (100%)	135 (100%)	405 (100%)
	No	0 (0.0%)	0 (0.0%)	0 (0.0%)	0 (0.0%)
Type of healthcare provider for pregnancy follow-up	Public sector	58 (43.0%)	48 (35.6%)	58 (43.0%)	164 (40.5%)
	Private sector	44 (32.6%)	39 (28.9%)	48 (35.6%)	131 (32.3%)
	Combination of both	33 (24.4%)	48 (35.6%)	29 (21.5%)	110 (27.2%)
Season of delivery	Spring	28 (20.7%)	31 (23.0%)	29 (21.5%)	88 (21.7%)
	Summer	42 (31.1%)	46 (34.1%)	36 (26.7%)	124 (30.6%)
	Autumn	32 (23.7%)	23 (17.0%)	25 (18.5%)	80 (19.8%)
	Winter	33 (24.4%)	35 (25.9%)	45 (33.3%)	113 (27.9%)

**Table 2:** Vaccination status against influenza and pertussis around pregnancy, by year.

Vaccine	Vaccination Status	2022	2023	2024	Total	*p*
		*n* (%)	*n* (%)	*n* (%)	*n* (%)	
Influenza	No vaccination	65 (48.1%)	74 (54.8%)	73 (54.1%)	212 (52.3%)	0.808
	During pregnancy	50 (37.0%)	45 (33.3%)	46 (34.1%)	141 (34.8%)	
	Postpartum	20 (14.8%)	16 (11.9%)	16 (11.9%)	52 (12.8%)	
Pertussis	No vaccination	97 (71.9%)	83 (61.5%)	61 (45.2%)	241 (59.5%)	<0.001
	During pregnancy	22 (16.3%)	32 (23.7%)	44 (32.6%)	98 (24.2%)	
	Postpartum	16 (11.9%)	20 (14.8%)	30 (22.2%)	66 (16.3%)	

For each vaccine, a composite variable with three categories (“No vaccination”, “During pregnancy”, and “Postpartum”) was created based on responses regarding vaccination during pregnancy and after delivery. The *p*-value (3 years) was calculated using the chi-square (χ^2^) test of independence to compare the distribution of categories across the years 2022, 2023, and 2024.

**Table 3:** Reasons for non-vaccination of mothers against influenza and pertussis.

Reasons for Non-Vaccination	2022	2023	2024	Total
	*n* (%)	*n* (%)	*n* (%)	*n* (%)
Influenza				
Safety concerns/possible side effects	8 (12.3%)	13 (17.6%)	17 (23.3%)	38 (17.9%)
Hesitancy or negative attitude toward vaccination	8 (12.3%)	4 (5.4%)	5 (6.8%)	17 (8.0%)
Considered unnecessary	49 (75.4%)	57 (77.0%)	51 (69.9%)	157 (74.1%)
Pertussis				
Safety concerns/possible side effects	3 (3.1%)	9 (10.8%)	7 (11.5%)	19 (7.9%)
Hesitancy or negative attitude toward vaccination	10 (10.3%)	4 (4.8%)	7 (11.5%)	21 (8.7%)
Considered unnecessary	84 (86.6%)	70 (84.3%)	47 (77.0%)	201 (83.4%)

**Table 4:** Vaccination of adult household contacts (father/close caregivers) and siblings of the neonate (where applicable) against influenza and pertussis.

Contact Vaccination	2022	2023	2024	Total
	*n* (%)	*n* (%)	*n* (%)	*n* (%)
**Influenza—Adult contacts**				
All	49 (36.3%)	44 (32.6%)	48 (35.6%)	141 (34.8%)
Father only	10 (7.4%)	9 (6.7%)	12 (8.9%)	31 (7.7%)
None	57 (42.2%)	64 (47.4%)	67 (49.6%)	188 (46.4%)
All except the father	19 (14.1%)	18 (13.3%)	8 (5.9%)	45 (11.1%)
**Influenza—Siblings**				
Yes	42 (53.8%)	29 (45.3%)	41 (55.4%)	112 (51.9%)
No	36 (46.2%)	35 (54.7%)	33 (44.6%)	104 (48.1%)
**Pertussis—Adult contacts**				
All	6 (4.4%)	13 (9.6%)	28 (20.7%)	47 (11.6%)
Father only	21 (15.6%)	27 (20.0%)	33 (24.4%)	81 (20.0%)
None	107 (79.3%)	94 (69.6%)	73 (54.1%)	274 (67.7%)
All except the father	1 (0.7%)	1 (0.7%)	1 (0.7%)	3 (0.7%)

**Table 5:** Reasons for non-vaccination of adult household contacts (when not all were vaccinated) and siblings against influenza and pertussis.

Reasons for Non-Vaccination	2022	2023	2024	Total
	*n* (%)	*n* (%)	*n* (%)	*n* (%)
**Influenza—Adult contacts**				
Safety concerns/possible side effects	2 (2.3%)	3 (3.3%)	5 (5.7%)	10 (3.8%)
Hesitancy or negative attitude toward vaccination	10 (11.6%)	13 (14.3%)	15 (17.2%)	38 (14.4%)
Considered unnecessary	74 (86.0%)	75 (82.4%)	67 (77.0%)	216 (81.8%)
**Influenza—Siblings**				
Safety concerns/possible side effects	3 (8.3%)	6 (17.1%)	9 (27.3%)	18 (17.3%)
Parental hesitancy or negative attitude toward vaccines	1 (2.8%)	1 (2.9%)	2 (6.1%)	4 (3.8%)
Considered unnecessary by parents	32 (88.9%)	28 (80.0%)	22 (66.7%)	82 (78.8%)
**Pertussis—Adult contacts**				
Safety concerns/possible side effects	0 (0.0%)	5 (4.1%)	2 (1.9%)	7 (2.0%)
Hesitancy or negative attitude toward vaccination	10 (7.8%)	6 (4.9%)	14 (13.1%)	30 (8.4%)
Considered unnecessary	119 (92.2%)	111 (91.0%)	91 (85.0%)	321 (89.7%)

**Table 6:** Adherence to recommendations of the NICU staff regarding vaccination against influenza and pertussis in subsequent pregnancies.

Compliance to Recommendations	2022	2023	2024	Total
	*n* (%)	*n* (%)	*n* (%)	*n* (%)
Yes	11 (40.7%)	7 (35.0%)	4 (40.0%)	22 (38.6%)
No	13 (48.1%)	9 (45.0%)	5 (50.0%)	27 (47.4%)
One of the two vaccines	3 (11.1%)	4 (20.0%)	1 (10.0%)	8 (14.0%)

**Table 7:** Binary logistic regression analysis of factors associated with influenza vaccination.

Variable	OR (95% CI)	*p*
High-risk group for influenza/COVID-19 (Yes vs. No)	1.26 (0.61–2.58)	0.533
Education level: Postgraduate/Doctoral	1.50 (0.76–2.94)	0.244
Education level: Primary	1.52 (0.59–3.94)	0.384
Education level: Tertiary	2.36 (1.47–3.80)	**<0.001**
Age group: 30–34 years	0.56 (0.29–1.08)	0.085
Age group: ≤25 years	0.24 (0.10–0.60)	**0.002**
Age group: ≥35 years	0.88 (0.47–1.64)	0.686

Outcome: binary variable indicating whether the woman received the influenza vaccine (during pregnancy and/or postpartum). Independent variables: educational level (reference category: secondary education), age group (reference category: 26–29 years), and membership in a high-risk group for influenza (Yes/No).

**Table 8:** Binary logistic regression analysis of factors associated with pertussis vaccination.

Variable	OR (95% CI)	*p*
High-risk group for influenza/COVID-19 (Yes vs. No)	0.67 (0.31–1.45)	0.305
Education level: Postgraduate/Doctoral	4.25 (2.02–8.94)	**<0.001**
Education level: Primary	0.95 (0.34–2.69)	0.928
Education level: Tertiary	1.51 (0.93–2.45)	0.098
Age group: 30–34 years	1.13 (0.57–2.25)	0.721
Age group: ≤25 years	0.50 (0.20–1.24)	0.138
Age group: ≥35 years	1.19 (0.62–2.28)	0.595

Outcome: binary variable indicating whether the woman received the pertussis vaccine (during pregnancy and/or postpartum). Independent variables: educational level (reference category: secondary education), age group (reference category: 26–29 years), and membership in a high-risk group for influenza (Yes/No).

## Data Availability

Data supporting the reported results, including the anonymized database and the study questionnaire, are available from the corresponding author upon reasonable request.

## Supplementary Materials

The following supporting information can be downloaded at: https://www.mdpi.com/article/10.3390/vaccines14070637/s1, File S1: Study Questionnaire.

## Abbreviations

The following abbreviations are used in this manuscript:

NICU	Neonatal Intensive Care Unit
WHO	World Health Organization
CDC	Centers for Disease Control and Prevention
