# Supplementary material for: Vaccination Coverage Among Mothers and Close Contacts of Neonates Hospitalized in NICU in Southern Greece: A Cocooning Strategy Approach
Source: Vaccines (Basel). 2026 Jul 20;14(7):637. doi: 10.3390/vaccines14070637 (PMC13416915; doi:10.3390/vaccines14070637)
Supplement: Supplementary file 1 [file vaccines-14-00637-s001.zip › vaccines-4422380-supplementary.pdf]

QUESTIONNAIRE

A. DEMOGRAPHIC INFORMATION

Participant Code:
Contact Telephone Number:

1. Age Group:
- ☐ ≤25 years
- ☐ 26–29 years
- ☐ 30–34 years
- ☐ ≥35 years

2. Nationality:
- ☐ Greek
- ☐ Albanian
- ☐ Bulgarian
- ☐ Other

3. Educational Level:
- ☐ Primary education
- ☐ Secondary education
- ☐ Tertiary (university) education
- ☐ Postgraduate or Doctoral studies

4. What is your occupation?
……………………………………………………………………………………

5. Marital Status:
- ☐ Married
- ☐ Single
- ☐ Divorced
- ☐ Widowed

6. Number of Children:
- ☐ 1
- ☐ 2
- ☐ 3
- ☐ 4
- ☐ ≥5

7. Do you belong to a high-risk group for influenza vaccination?
- ☐ Yes
- ☐ No

8. Do you have health insurance?
- ☐ Yes
- ☐ No

9. Method of Conception:
- ☐ Natural conception
- ☐ Assisted reproductive technology

10. Did you receive medical care during your pregnancy?
- ☐ Yes
- ☐ No

11. If yes, was your prenatal care provided in:
- ☐ Private healthcare sector
- ☐ Public healthcare sector

☐ Both private and public healthcare sector

12. Season of Delivery:
☐ Winter
☐ Spring
☐ Summer
☐ Autumn

B. INFLUENZA VACCINE

1. Did you receive the influenza vaccine during pregnancy?
☐ Yes
☐ No

2. If yes, who recommended it?
☐ Obstetrician/Gynecologist
☐ Internist or General Practitioner
☐ Pediatrician
☐ Pharmacist
☐ Other

3. Did you receive the influenza vaccine during the postpartum period?
☐ Yes
☐ No

4. If no, why were you not vaccinated?
☐ I had concerns about the vaccine's safety for the fetus/newborn.
☐ I had concerns about possible side effects for myself.
☐ I have reservations or a negative attitude toward vaccinations in general.
☐ I did not consider it necessary.

5. Were the baby's father and/or other close caregivers (e.g., grandparents) vaccinated?
☐ Yes, all of them.
☐ Only the father.
☐ No, none of them.

6. If not, why were they not vaccinated?
☐ Concerns about the vaccine's safety or possible side effects.
☐ Reservations or a negative attitude toward vaccinations in general.
☐ They did not consider it necessary.

7. Were the newborn's siblings vaccinated?
☐ Yes
☐ No

8. If not, why were they not vaccinated?
☐ Concerns about the vaccine's safety or possible side effects.
☐ Parents had reservations or a negative attitude toward vaccinations in general.
☐ The parents did not consider it necessary.

C. PERTUSSIS VACCINE

1. Did you receive the pertussis vaccine during pregnancy?
☐ Yes
☐ No

2. If yes, who recommended it?
☐ Obstetrician/Gynecologist
☐ Internist or General Practitioner
☐ Pediatrician
☐ Pharmacist
☐ Other

3. Did you receive the pertussis vaccine during the postpartum period?
☐ Yes
☐ No

4. If no, why were you not vaccinated?
☐ I had concerns about the vaccine's safety for the fetus/newborn.
☐ I had concerns about possible side effects for myself.
☐ I have reservations or a negative attitude toward vaccinations in general.
☐ I did not consider it necessary.

5. Were the baby's father and/or other close caregivers (e.g., grandparents) vaccinated?
☐ Yes, all of them.
☐ Only the father.
☐ No, none of them.

6. If not, why were they not vaccinated?
☐ Concerns about the vaccine's safety or possible side effects.
☐ Reservations or a negative attitude toward vaccinations in general.
☐ They did not consider it necessary.

D. SUBSEQUENT PREGNANCIES

1. Did you have another child in the following years?
☐ Yes
☐ No

2. If yes, did you follow the recommendations provided by the NICU staff regarding influenza and pertussis vaccinations?
☐ Yes
☐ No
